# Lactated Ringer’s Versus Normal Saline in Early-Phase Acute Pancreatitis: A Comparative Study of Inflammatory Markers and Clinical Outcomes

**DOI:** 10.7759/cureus.107272

**Published:** 2026-04-17

**Authors:** Zawar Ahmad, Zeeshan Umar, Saleem Shah, Saeed Sarwar, Abbas Khan, Hafsah Iqbal, Sundas Safdar, Malik Muaz Iqbal, Zahid Shah, Moiz Inam Khan

**Affiliations:** 1 Trauma and Orthopedics, Advance Medical Complex Shergarh, Mardan, PAK; 2 Trauma and Orthopedics, Kettering General Hospital, Kettering, GBR; 3 Emergency Medicine, Pak Emirates Military Hospital (PEMH), Rawalpindi, PAK; 4 Internal Medicine, Mardan Medical Complex, Mardan, PAK; 5 General Surgery, Khyber Teaching Hospital, Peshawar, PAK; 6 Internal Medicine, Saidu Group of Teaching Hospital, Saidu Sharif, PAK; 7 Internal Medicine, Shaukat Khanum Memorial Cancer Hospital and Research Centre, Peshawar, PAK; 8 Diagnostic Radiology, Lady Reading Hospital, Peshawar, PAK; 9 Internal Medicine, Lady Reading Hospital, Peshawar, PAK; 10 Internal Medicine, Shifa Hospital Swat, Saidu Sharif, PAK; 11 Accident and Emergency Medicine, Medical Teaching Institution District Headquarters Teaching Hospital, Dera Ismail Khan, PAK

**Keywords:** acute pancreatitis, c-reactive protein, fluid resuscitation, lactated ringer’s, normal saline, systemic inflammatory response syndrome

## Abstract

Background and aim

Early fluid resuscitation is essential for reducing inflammation and improving outcomes in acute pancreatitis. The primary objective of this study was to compare changes in inflammatory markers, particularly C-reactive protein, between lactated Ringer’s (LR) and normal saline (NS) in early acute pancreatitis. Secondary objectives included comparison of early clinical outcomes (persistent systemic inflammatory response syndrome (SIRS), ICU admission, hospital stay, complications, and mortality) and late outcomes at six months.

Methodology

This prospective comparative study was conducted from July 2023 to June 2025. According to hospital fluid-procedure guidelines, 164 patients who presented within 24 h of the beginning of symptoms were included and given either LR (n=82) or NS (n=82). At admission and 48-72 h later, baseline demographics, clinical characteristics, and laboratory indicators, such as hematocrit, white blood cell (WBC) count, and C-reactive protein (CRP), were documented. In-hospital mortality, length of hospital stay, intensive care unit (ICU) admission, persistence of SIRS, and systemic and local sequelae were among the early clinical outcomes that were recorded. Over a six-month period, late outcomes were assessed, including the development of pancreatic pseudocysts, necrosis-related sequelae, readmission, and death. Independent t-tests, chi-square tests, and multivariate logistic regression were among the statistical analyses performed.

Results

At 48-72 h, CRP was significantly lower in the LR group (68.21±24.32 mg/L) than in NS (79.54±27.12 mg/L, p=0.01), and WBC counts declined more with LR (11.08±2.98×10³/µL versus 12.76±3.15×10³/µL, p=0.002). SIRS persistence >48 h occurred in 12 LR patients (14.63%) versus 22 NS patients (26.83%, p=0.04), while mean hospital stay was shorter with LR (6.12±2.14 versus 7.46±2.68 days, p=0.001). ICU admission, complications, and in-hospital mortality were lower in LR but not statistically significant. Late outcomes showed non-significant trends favoring LR.

Conclusion

Lactated Ringer’s solution provides superior early anti-inflammatory effects and improved clinical outcomes compared to normal saline in early acute pancreatitis. Patients receiving lactated Ringer’s demonstrated greater reductions in inflammatory markers, including C-reactive protein and white blood cell counts, as well as reduced persistence of systemic inflammatory response syndrome and shorter hospital stays. Although trends toward reduced complications and mortality were observed, these did not reach statistical significance. Further large-scale randomized controlled trials are warranted to validate these findings and guide clinical practice.

## Introduction

Acute pancreatitis is an inflammatory disorder of the pancreas, with a clinical spectrum ranging from mild, self-limiting disease to severe necrotizing pancreatitis characterized by systemic inflammatory response syndrome (SIRS), organ dysfunction, and increased mortality [1,2]. Globally, it is an increasingly growing disease, which is mostly caused by gallstone disease, alcoholism, hypertriglyceridemia, and metabolic disorders [3]. It is important to detect and manage the disease early in order to avoid further development of the disease and minimize complications [4].

Pathophysiology of acute pancreatitis entails untimely activation of pancreatic enzymes, which causes self-digestion, cellular injuries of acinar cells, and the discharge of pro-inflammatory cytokines. This inflammatory pathway may spread beyond the pancreas, causing systemic problems [5]. The initial 24-48 h of disease progression represent a critical therapeutic window, and appropriate fluid resuscitation plays a key role in determining pancreatic perfusion, systemic inflammation, and overall disease severity [6,7]. Aggressive intravenous fluid resuscitation is one of the key principles of early management. Fluid replacement restores intravascular volume, enhances pancreatic microcirculation, reduces ischemia, and mitigates inflammation [8].

Fluid resuscitation is usually done with crystalloid solutions, the most widely used being the normal saline (0.9% sodium chloride) and the lactated Ringer's solution [9]. However, as a commonly used and inexpensive resuscitative fluid, normal saline has been associated with hyperchloremic metabolic acidosis, particularly when administered in large volumes (e.g., >3-4 L within the first 24 h) during aggressive resuscitation, which may adversely affect systemic inflammation and renal perfusion [10]. Conversely, lactated Ringer's solution is a balanced crystalloid solution consisting of sodium, potassium, calcium, chloride, and lactate that is a precursor of the bicarbonate [11]. Emerging evidence suggests that balanced crystalloids may reduce the inflammatory response and improve clinical outcomes in acute pancreatitis. However, current studies report mixed and sometimes contradictory findings, particularly regarding their effects on inflammatory markers and disease progression [12].

C-reactive protein (CRP) and white blood cell (WBC) count are established inflammatory markers, while hematocrit serves as a marker of hemoconcentration and intravascular volume status, helping to assess disease severity and response to therapy in acute pancreatitis [13]. Following these indicators, as well as clinical outcome measures such as the persistence of SIRS, hospitalization duration, intensive care requirement, and complication rate, provides information on the quality of resuscitation measures [14]. Since the preferred option of crystalloid in the early phase of acute pancreatitis remains a debatable issue, the comparative analysis of lactated Ringer's and normal saline should be conducted to shed light on their effects on systemic inflammation and patient outcomes.

## Materials and methods

Study design and setting

This was a prospective, non-randomized comparative study conducted at Lady Reading Hospital, Peshawar, from July 2023 to June 2025. Consecutive adult patients (≥18 years) presenting with early-phase acute pancreatitis (within 24 h of symptom onset) were enrolled to ensure early-phase management, maintain uniformity in treatment, and minimize potential confounders. Patients were allocated to receive either lactated Ringer’s (LR) solution or normal saline (NS) according to the standard fluid protocol of the admitting unit. Both groups followed a standardized, goal-directed fluid resuscitation regimen consistent with the International Association of Pancreatology Revised Guidelines on Acute Pancreatitis 2025 [15]. Intravenous hydration was initiated within the first 24 h, with an initial bolus of 10-20 mL/kg over 60-90 min for patients with clinical evidence of hypovolemia, followed by a maintenance infusion of 1.5-3 mL/kg/h, adjusted based on dynamic clinical parameters including heart rate, mean arterial pressure, urine output (target 0.5 mL/kg/h), hematocrit trends, blood urea nitrogen, and overall hemodynamics. Fluid rates were reviewed at 6-12 h to prevent both under-resuscitation and fluid overload.

Inclusion and exclusion criteria

Patients aged ≥18 years with a diagnosis of acute pancreatitis were included in this study. The diagnostic criteria required at least two of the following characteristics: upper abdominal pain suggestive of pancreatitis, serum amylase and/or lipase levels at least three times the upper limit of normal, or radiological findings consistent with acute pancreatitis. Patients were included only if they presented within 24 h of symptom onset to ensure early-phase management, maintain uniformity in treatment, and minimize potential confounding variables. The patients who had chronic pancreatitis, pancreatic malignancy, advanced hepatic, renal or cardiac failure at admission, non-pancreatic sepsis, more than 24 h of previous fluid resuscitation, or pregnancy were excluded.

Sample size

The decrease in C-reactive protein (CRP) levels was the main outcome measure used to determine the sample size for the comparison of two independent means. Assuming a mean difference of 8 mg/L and a pooled standard deviation of 18 mg/L, the minimum necessary sample size was calculated to be 79 patients per group, for a total of 158 participants, with a 95% confidence level and 80% statistical power. The sample size was expanded to 182 patients to account for an expected 15% loss to follow-up. After incomplete follow-up and attrition, 164 patients comprised the final study sample.

Data collection

Baseline demographic and clinical variables, including age, gender, body mass index, comorbidities, etiology of pancreatitis, and vital signs at admission, were recorded. Laboratory parameters, including complete blood count, hematocrit, serum electrolytes, renal function tests, and C-reactive protein (CRP), were measured at admission and repeated at a standardized time point of 48 h (±4 h) for all patients to ensure consistency between the LR and NS groups. Disease severity at admission was assessed using the Bedside Index for Severity in Acute Pancreatitis (BISAP) score [16]. Clinical outcomes evaluated included in-hospital mortality, length of hospital stay, ICU admission, development of local or systemic complications, and persistence of systemic inflammatory response syndrome (SIRS). Patients were followed for six months after discharge to assess late outcomes, such as pancreatic pseudocyst formation, necrosis-related sequelae, readmissions, and mortality.

Statistical analysis

Statistical Package for Social Sciences (SPSS) version 26 (Armonk, NY: IBM Corp.) was used to enter and analyze the data. Whereas categorical data were displayed as frequencies and percentages, continuous variables were represented as mean±standard deviation. The two groups' mean levels of inflammatory markers were compared using the independent sample t-test. Categorical variables were subjected to the chi-square test. In order to account for potential confounding factors such as age, comorbidities, and baseline BISAP score, multivariate logistic regression analysis was also conducted. Statistical significance was defined as a p-value <0.05.

Ethical approval

**Figure 1:**
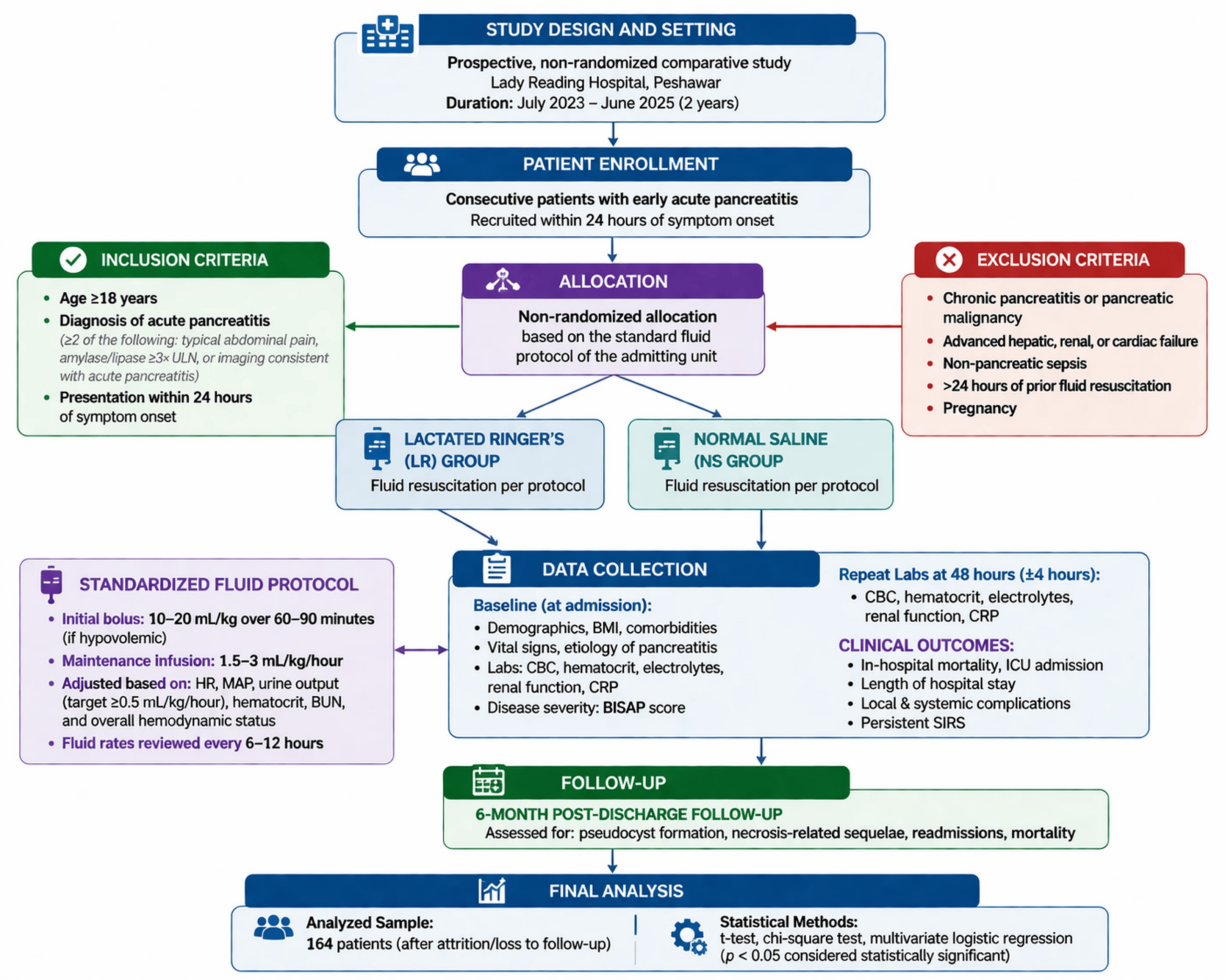
Study flowchart for early acute pancreatitis. MAP: mean arterial pressure; BUN: blood urea nitrogen; BISAP: Bedside Index for Severity in Acute Pancreatitis; SIRS: systemic inflammatory response syndrome

The Institutional Review Board of Lady Reading Hospital Medical Teaching Institution, Peshawar, approved the study. All participants provided written informed consent. The study was carried out in compliance with the Declaration of Helsinki's ethical guidelines, and patient information was kept private. The study flowchart for early acute pancreatitis is summarized in Figure 1.

## Results

The LR and NS groups were comparable in terms of baseline demographics and clinical characteristics (Table 1). The mean age was 45.62±12.45 years in the LR group and 46.17±11.88 years in the NS group (p=0.72). Gender distribution was similar, with 58.54% males in the LR group and 60.98% in the NS group. Mean BMI was 26.34±3.89 kg/m² for LR and 26.81±4.12 kg/m² for NS (p=0.48). Etiology of pancreatitis was balanced between groups, with gallstones accounting for 43.90% versus 41.46%, alcohol 24.39% versus 26.83%, hypertriglyceridemia 18.29% versus 17.07%, and idiopathic 13.41% versus 14.63% in the LR and NS groups, respectively. Comorbidities and admission vital signs, including heart rate and mean arterial pressure, showed no significant differences. Bedside Index for Severity in Acute Pancreatitis (BISAP) score ≥3 was observed in 12.20% of LR patients and 14.63% of NS patients (p=0.65), indicating similar baseline disease severity.

**Table 1:** Baseline characteristics and comorbidities of study participants. BISAP: Bedside Index for Severity in Acute Pancreatitis; LR: lactated Ringer’s; NS: normal saline; MAP: mean arterial pressure

Categories	Characteristics	LR group (n=82)	NS group (n=82)	p-Value
Demographics	Age (years), mean±SD	45.62±12.45	46.17±11.88	0.72
	Male, n (%)	48 (58.54)	50 (60.98)	0.78
	Female, n (%)	34 (41.46)	32 (39.02)	0.78
	BMI (kg/m²), mean±SD	26.34±3.89	26.81±4.12	0.48
Etiology of pancreatitis	Gallstones, n (%)	36 (43.90)	34 (41.46)	0.74
	Alcohol, n (%)	20 (24.39)	22 (26.83)	0.71
	Hypertriglyceridemia, n (%)	15 (18.29)	14 (17.07)	0.84
	Idiopathic, n (%)	11 (13.41)	12 (14.63)	0.82
Comorbidities	Diabetes mellitus, n (%)	18 (21.95)	20 (24.39)	0.71
	Hypertension, n (%)	14 (17.07)	15 (18.29)	0.84
	Cardiovascular disease, n (%)	5 (6.10)	6 (7.32)	0.75
Vital signs at admission	Heart rate (bpm), mean±SD	92.48±10.35	93.76±11.12	0.54
	MAP (mmHg), mean±SD	84.32±7.28	83.79±7.55	0.66
Disease severity	BISAP score ≥3, n (%)	10 (12.20%)	12 (14.63%)	0.65

Laboratory markers at admission were similar in both groups (Table 2). Mean CRP at admission was 102.45±32.18 mg/L in the LR group versus 101.76±31.64 mg/L in the NS group (p=0.88). At 48-72 h, CRP decreased to 68.21±24.32 mg/L in the LR group, significantly lower than 79.54±27.12 mg/L in the NS group (p=0.01). WBC counts also decreased more in the LR group (11.08±2.98×10³/µL) compared to NS (12.76±3.15×10³/µL, p=0.002). Hematocrit dropped to 41.26±3.12% in LR versus 42.89±3.21% in NS (p=0.004). Serum creatinine at 48-72 h was lower in LR (0.98±0.19 mg/dL) compared to NS (1.08±0.21 mg/dL, p=0.03). Sodium and potassium levels remained comparable. These results suggest more favorable inflammatory and renal profiles with LR.

**Table 2:** Laboratory parameters at admission and 48-72 h. *P<0.05 indicates a statistically significant difference. LR: lactated Ringer’s; NS: normal saline

Parameters	Timepoint	LR group (n=82)	NS group (n=82)	p-Value
C-reactive protein (mg/L)	Admission	102.45±32.18	101.76±31.64	0.88
	48-72 h	68.21±24.32	79.54±27.12	0.01*
WBC (×10³/µL)	Admission	14.12±3.45	14.35±3.62	0.68
	48-72 h	11.08±2.98	12.76±3.15	0.002*
Hematocrit (%)	Admission	44.18±3.56	44.75±3.48	0.31
	48-72 h	41.26±3.12	42.89±3.21	0.004*
Serum creatinine (mg/dL)	Admission	1.02±0.21	1.04±0.23	0.62
	48-72 h	0.98±0.19	1.08±0.21	0.03*
Serum sodium (mmol/L)	Admission	138.6±3.2	138.2±3.5	0.41
Serum potassium (mmol/L)	Admission	4.1±0.4	4.0±0.5	0.29

In Table 3, early clinical outcomes during hospitalization showed that SIRS persistence beyond 48 h occurred in 12 (14.63%) patients in the lactated Ringer’s (LR) group compared with 22 (26.83%) patients in the normal saline (NS) group, which was statistically significant (p=0.04). ICU admission was required in eight (9.76%) LR patients versus 15 (18.29%) NS patients (p=0.09). The mean length of hospital stay was significantly shorter in the LR group (6.12±2.14 days) than in the NS group (7.46±2.68 days; p=0.001). Local complications occurred in six (7.32%) LR patients and 11 (13.41%) NS patients, while systemic complications were observed in four (4.88%) LR patients versus nine (10.98%) NS patients; neither reached statistical significance. In-hospital mortality was low, with one (1.22%) patient in the LR group and three (3.66%) patients in the NS group (p=0.31).

**Table 3:** Early and late clinical outcomes with subgroup analysis by etiology. *P<0.05 indicates a statistically significant difference. Subgroup data shown only for outcomes where trends or significance exist. “-” indicates data not broken down due to small numbers or lack of statistical relevance. SIRS: systemic inflammatory response syndrome; LR: lactated Ringer’s; NS: normal saline

Outcome category	Outcomes	LR group (n=82)	NS group (n=82)	p-Value	Gallstones LR/NS	Alcohol LR/NS	Hypertriglyceridemia LR/NS	Idiopathic LR/NS
Early outcomes (during hospitalization)	SIRS persistence >48 h, n (%)	12 (14.63)	22 (26.83)	0.04*	4/9	3/6	2/3	3/4
	ICU admission, n (%)	8 (9.76%)	15 (18.29%)	0.09	-	-	-	-
	Length of hospital stay (days), mean±SD	6.12±2.14	7.46±2.68	0.001*	5.9±1.9/7.4±2.5	6.3±2.0/7.5±2.7	6.1±2.1/7.2±2.8	6.4±2.2/7.5±2.9
	Local complications, n (%)	6 (7.32)	11 (13.41)	0.17	2/5	1/2	1/2	1/2
	Systemic complications, n (%)	4 (4.88)	9 (10.98)	0.12	-	-	-	-
	In-hospital mortality, n (%)	1 (1.22)	3 (3.66)	0.31	-	-	-	-
Late outcomes (6-month follow-up)	Pancreatic pseudocyst formation, n (%)	2 (2.47)	5 (6.33)	0.23	-	-	-	-
	Necrosis-related sequelae, n (%)	1 (1.23)	3 (3.80)	0.30	-	-	-	-
	Readmission within 6 months, n (%)	3 (3.70)	7 (8.86)	0.15	-	-	-	-
	Mortality within 6 months, n (%)	1 (1.23)	2 (2.53)	0.56	-	-	-	-

During the six-month follow-up, late outcomes showed pancreatic pseudocyst formation in two (2.47%) LR patients versus five (6.33%) NS patients, and necrosis-related sequelae in one (1.23%) LR patient versus three (3.80%) NS patients, with no statistical significance. Readmission rates were 3.70% in the LR group compared to 8.86% in the NS group, and mortality during follow-up was 1.23% in the LR group versus 2.53% in the NS group, again not statistically significant, although there was a trend toward fewer complications in the LR group.

After adjusting for age, comorbidities, and baseline BISAP score, LR was associated with significantly lower odds of SIRS persistence >48 h (adjusted odds ratio {AOR}: 0.48, 95% CI: 0.24-0.95, p=0.036) and lower odds of local or systemic complications (AOR: 0.57, 95% CI: 0.22-1.48, p=0.25) (Table 4). A higher BISAP score (≥3) increased the risk of SIRS persistence (AOR: 2.12, p=0.047) and complications (AOR: 3.01, p=0.044). ICU admission and other outcomes did not reach statistical significance, confirming that LR improved early inflammatory and clinical outcomes after controlling for confounders.

**Table 4:** Multivariate logistic regression analysis of fluid type on key clinical outcomes. *P<0.05 indicates a statistically significant difference. AOR: adjusted odds ratio; BISAP: Bedside Index for Severity in Acute Pancreatitis; SIRS: systemic inflammatory response syndrome; LR: lactated Ringer’s; NS: normal saline

Outcome	Variables	AOR	95% confidence interval	p-Value
SIRS persistence >48 h	LR versus NS	0.48	0.24-0.95	0.036*
	Age (per year increase)	1.02	0.99-1.05	0.18
	Comorbidities (yes versus no)	1.34	0.67-2.68	0.41
	BISAP ≥3 versus <3	2.12	1.01-4.45	0.047*
ICU admission	LR versus NS	0.52	0.21-1.27	0.15
	Age	1.01	0.97-1.05	0.62
	Comorbidities	1.45	0.56-3.72	0.44
	BISAP ≥3 versus <3	2.75	0.97-7.79	0.06
Local or systemic complications	LR versus NS	0.57	0.22-1.48	0.25
	Age	1.03	0.99-1.06	0.12
	Comorbidities	1.61	0.68-3.79	0.28
	BISAP ≥3 versus <3	3.01	1.03-8.79	0.044*

## Discussion

In the present study, patients with early acute pancreatitis who were resuscitated with LR solution demonstrated a greater reduction in inflammatory markers compared to those who received NS. Specifically, mean CRP levels at 48-72 h were significantly lower in the LR group (68.21±24.32 mg/L) compared to the NS group (79.54±27.12 mg/L, p=0.01). Similarly, WBC counts showed a greater reduction in the LR group (11.08±2.98×10³/µL versus 12.76±3.15×10³/µL, p=0.002). These results are in line with the existing evidence that suggests that LR has an anti-inflammatory effect. Indicatively, a randomized controlled trial noted that LR had a significant effect on reducing CRP levels and systemic inflammatory response syndrome than NS in the acute stage of pancreatitis patients (e.g., CRP: 51.5 mg/L versus 104 mg/L; p=0.02), which indicates the use of fluids in balancing the inflammatory process [17]. Further, a large systematic review and meta-analysis also found that LR was linked to a much lower CRP at 48 h (SMD: -3.91; p<0.00001) but less so in the later points, which argues in favor of the early anti-inflammatory effect in this study [18].

As far as early clinical outcomes were concerned, LR resuscitation was linked to a significantly lesser persistence of SIRS, lasting more than 48 h (14.63% versus 26.83%, p=0.04), as well as a shorter hospital stay (6.12±2.14 versus 7.46±2.68 days, p=0.001). These findings are consistent with wider-ranging evidence that balanced crystalloids like LR can reduce disease progression and the need for ICU support compared to NS; meta-analytic evidence showed that patients who received balanced fluids had lower chances of developing moderately severe or severe acute pancreatitis and decreased ICU hospitalization [19]. In the same way, previous systematic reviews have identified reduced ICU admissions and shortened hospital stays in LR groups relative to NS, although SIRS at fixed early time intervals did not necessarily significantly differ between the groups [20].

The LR group had lower hospital mortality and other complications (local or systemic), though (the difference was not statistically significant) in the unadjusted analyses. These results are in line with meta-analytic findings in which mortality patterns favored LR but were not statistically significant, probably because of the low overall event rates and limited power in each trial [18]. Our multivariate analysis showed that LR had an independent odds reduction effect of persistent SIRS (adjusted odds ratio {AOR}: 0.48, p=0.036) and proposed a smaller odds of other complications with age, comorbidity, and baseline BISAP score adjustments. This has been supported by data showing that balanced crystalloids have the potential to reduce the risk of ICU hospitalization and local complications by a significant margin compared with NS [9].

There were non-significant trends towards a few events in the LR group in late outcomes at six months, such as pseudocyst formation, necrosis-related sequelae, readmissions, and mortality. The long-term sequelae differences between LR and NS have not been consistently reported in past research, which could be attributed to the multifactorial nature of post-discharge complications on top of early fluid choice and the inconsistent nature of follow-up protocols used in various studies. Comprehensively, our results have added to the body of literature that balanced crystalloid solutions like lactated Ringer's have a positive anti-inflammatory effect and good early clinical outcomes in acute pancreatitis in comparison to normal saline.

Limitations and strengths of the study

This study has several strengths. It prospectively compared lactated Ringer’s (LR) solution and normal saline (NS) for the management of acute pancreatitis using a standardized fluid resuscitation protocol, ensuring consistent early-phase management and systematic monitoring of inflammatory markers (CRP, WBC, hematocrit) and clinical outcomes. The six-month follow-up allowed evaluation of late complications, including pseudocyst formation, necrosis-related sequelae, readmissions, and mortality, providing a comprehensive assessment beyond hospitalization. Adjustment for potential confounders, including age, comorbidities, and baseline BISAP score using multivariate logistic regression, strengthened the reliability of the findings. Furthermore, an exploratory subgroup analysis by etiology (gallstones, alcohol, hypertriglyceridemia, and idiopathic) demonstrated consistent trends favoring LR across different causes of pancreatitis, adding further support to the observed associations.

Several limitations should also be acknowledged. This study was non-randomized, and allocation based on the admitting unit’s protocol may have introduced selection bias. As a single-center study, the generalizability of findings may be limited. Although the sample size was adequate for detecting differences in inflammatory markers, it may have been underpowered to detect rare outcomes such as mortality. Some post-discharge variables, such as fluid responsiveness, timing of nutritional support, and adjunctive therapies, were not assessed and could have influenced outcomes. Although subgroup analysis was performed post hoc, the relatively small numbers in each etiological subgroup limit statistical power and preclude definitive conclusions. Future multicenter, randomized controlled trials with larger sample sizes and standardized treatment protocols, including pre-specified etiological subgroup analyses, are warranted to confirm these associations and better define the role of fluid type across diverse patient populations.

## Conclusions

The findings of this study suggest that lactated Ringer’s solution may be associated with improved early anti-inflammatory effects and favorable trends in clinical outcomes compared to normal saline in patients with early acute pancreatitis. Patients receiving lactated Ringer’s demonstrated lower CRP and WBC levels at 48-72 h, reduced persistence of SIRS, and shorter hospital stays. Although trends toward reduced complications and mortality were observed, these did not reach statistical significance. Given the observational, non-randomized design and the potential influence of unmeasured confounders, these results should be interpreted cautiously. Larger, multicenter randomized controlled trials are warranted to confirm these associations and guide definitive clinical recommendations.
